# Exercise for Neuropathic Pain: A Systematic Review and Expert Consensus

**DOI:** 10.3389/fmed.2021.756940

**Published:** 2021-11-24

**Authors:** Yong-Hui Zhang, Hao-Yu Hu, Yuan-Chang Xiong, Changgeng Peng, Li Hu, Ya-Zhuo Kong, Yu-Ling Wang, Jia-Bao Guo, Sheng Bi, Tie-Shan Li, Li-Juan Ao, Chu-Huai Wang, Yu-Long Bai, Lei Fang, Chao Ma, Lin-Rong Liao, Hao Liu, Yi Zhu, Zhi-Jie Zhang, Chun-Long Liu, Guo-En Fang, Xue-Qiang Wang

**Affiliations:** ^1^Department of Sport Rehabilitation, Shanghai University of Sport, Shanghai, China; ^2^Department of Rehabilitation Medicine, Shanghai Shangti Orthopedic Hospital, Shanghai, China; ^3^Department of Pain Therapy, First Affiliated Hospital of Naval Medical University, Shanghai, China; ^4^The First Rehabilitation Hospital of Shanghai, Brain and Spinal Cord Innovation Research Center, School of Medicine, Advanced Institute of Translational Medicine, Tongji University, Shanghai, China; ^5^Institute of Psychology, Chinese Academy of Sciences, Beijing, China; ^6^Department of Rehabilitation Medicine, The Sixth Affiliated Hospital of Sun Yat-sen University, Guangzhou, China; ^7^The Second School of Clinical Medicine, Xuzhou Medical University, Xuzhou, China; ^8^Rehabilitation Medicine Centre, Chinese PLA General Hospital, Beijing, China; ^9^Department of Rehabilitation Medicine, The Affiliated Hospital of Qingdao University, Qingdao, China; ^10^School of Rehabilitation, Kunming Medical University, Kunming, China; ^11^Department of Rehabilitation Medicine, The First Affiliated Hospital, Sun Yat-sen University, Guangzhou, China; ^12^Department of Rehabilitation Medicine, Huashan Hospital, Fudan University, Shanghai, China; ^13^School of Rehabilitation Science, Shanghai University of T.C.M., Shanghai, China; ^14^Department of Rehabilitation, Sun Yat-sen Memorial Hospital, Sun Yat-sen University, Guangzhou, China; ^15^Department of Rehabilitation, Yixing JORU Rehabilitation Hospital, Wuxi, China; ^16^Department of Pain and Musculoskeletal Rehabilitation, The Fifth Affiliated Hospital of Zhengzhou University, Zhengzhou, China; ^17^Rehabilitation Therapy Center, Luoyang Orthopedic Hospital of Henan Province, Orthopedic Hospital of Henan Province, Luoyang, China; ^18^Clinical Medical College of Acupuncture, Moxibustion and Rehabilitation, Guangzhou University of Chinese Medicine, Guangzhou, China; ^19^Department of Gastrointestinal Surgery, First Affiliated Hospital of Naval Medical University, Shanghai, China

**Keywords:** exercise, training, neuropathic pain, chronic pain, expert consensus

## Abstract

**Background:** Neuropathic pain (NP), a severe and disruptive symptom following many diseases, normally restricts patients' physical functions and leads to anxiety and depression. As an economical and effective therapy, exercise may be helpful in NP management. However, few guidelines and reviews focused on exercise therapy for NP associated with specific diseases. The study aimed to summarize the effectiveness and efficacy of exercise for various diseases with NP supported by evidence, describe expert recommendations for NP from different causes, and inform policymakers of the guidelines.

**Design:** A systematic review and expert consensus.

**Methods:** A systematic search was conducted in PubMed. We included systematic review and meta-analysis, randomized controlled trials (RCTs), which assessed patients with NP. Studies involved exercise intervention and outcome included pain intensity at least. Physiotherapy Evidence Database and the Assessment of Multiple Systematic reviews tool were used to grade the quality assessment of the included RCTs and systematic reviews, respectively. The final grades of recommendation were based on strength of evidence and a consensus discussion of results of Delphi rounds by the Delphi consensus panel including 21 experts from the Chinese Association of Rehabilitation Medicine.

**Results:** Eight systematic reviews and 21 RCTs fulfilled all of the inclusion criteria and were included, which were used to create the 10 evidence-based consensus statements. The 10 expert recommendations regarding exercise for NP symptoms were relevant to the following 10 different diseases: spinal cord injury, stroke, multiple sclerosis, Parkinson's disease, cervical radiculopathy, sciatica, diabetic neuropathy, chemotherapy-induced peripheral neuropathy, HIV/AIDS, and surgery, respectively. The exercise recommended in the expert consensus involved but was not limited to muscle stretching, strengthening/resistance exercise, aerobic exercise, motor control/stabilization training and mind-body exercise (Tai Chi and yoga).

**Conclusions:** Based on the available evidence, exercise is helpful to alleviate NP intensity. Therefore, these expert consensuses recommend that proper exercise programs can be considered as an effective alternative treatment or complementary therapy for most patients with NP. The expert consensus provided medical staff and policymakers with applicable recommendations for the formulation of exercise prescription for NP. This consensus statement will require regular updates after five–ten years.

## Introduction

Neuropathic pain (NP) is defined as pain driven by a lesion or disease of the somatosensory nervous system (1, 2). Meanwhile, central or peripheral nerve lesions can lead to sensory loss in the corresponding body regions to the damaged central nervous part or in the innervation territory of injured peripheral nerve. Indeed, one of the most important features of NP is a complex combination of sensory loss and pain. It is well-known that NP is not only an exclusive symptom for patients with direct nervous injuries but also indirect nervous peripheral neuropathy. For instance, the incidence of NP is around 50% in patients with spinal cord injury and ~21% in patients suffering from diabetic neuropathy (3). Compared with males (5.7%), the female population is more likely to suffer from chronic NP (8%) (1). Additionally, the related more affected body regions are low back, neck, and extremities (4). The symptoms of NP, such as spontaneous pain, evoked pain, aftersensation, hyperalgesia, and referred pain, could seriously disturb patients' motor function and emotions and result in a low quality of life, anxiety, and depression. The current management of NP aim to control or ameliorate symptoms due to the difficulty of treating damaged nerve directly. However, traditional pharmacological treatment is not effective enough and may lead to cardiac conduction block, sedation, anticholinergic effects or opioid-related adverse effects (5, 6). Thus, non-pharmacological approaches, such as exercise, have gained the attention of physicians.

Exercise, a feasible and economical way, has been widely accepted as an effective treatment for musculoskeletal disorders. As a treatment, exercise refers to the physical activities aiming to correct impairment and improve physical and cognitive function, which can positively contribute to health (7). Normally, therapeutic exercise could be divided into various types, such as muscle stretching, strengthening/resistance exercise, aerobic exercise, motor control/stabilization training and mind-body exercise (8). Considering the benefits of exercise, such as blood glucose and blood lipid reduction, exercise-induced hypoalgesia and emotional improvement, it might be an effective way to prevent and treat NP (9, 10). The effectiveness of exercise training as a complementary therapy or interventional treatment for patients with NP has been previously reported and exercise program seems to be beneficial to the recovery of damaged peripheral nerve, the alleviation of pain symptoms, and the improvement of physical status (11). However, the distinct content of exercise, different intensities of training, and various frequencies of physical activities can produce different effects and influence on patients with NP. Similarly, NP from different causes have diverse characteristics and are likely to respond to exercise treatment differently. A proper exercise plan for the management of NP in patients has been a challenge for physicians and physiotherapists.

Several clinical guidelines, systematic reviews, and meta-analyses regarding to clinical therapies for some specific diseases with NP symptoms have been published (12–17). However, these guidelines and reviews more focus on pharmacological treatment; non-invasive treatments, such electrical and magnetic stimulation; and other non-pharmacological approaches, but not exercise. Although exercise has been reported as a safe and useful method to improve functions and relieve pain in patients with NP, few guidelines or expert consensus review exercise program as a treatment for different types of NP in detail. Therefore, the Chinese Association of Rehabilitation Medicine needs to establish an exercise program consensus for diseases with NP that could be applicable to physiotherapists.

The Chinese Association of Rehabilitation Medicine invited experts in physiotherapy, sports science, orthopedics, and sports medicine to develop evidence-based recommendations and expert consensus. This expert consensus aimed to: (1) summarize the effectiveness and efficacy of exercise for various diseases with NP supported by evidence; (2) describe evidence-based exercise recommendations for NP from different causes, including central and peripheral nerve damage; and (3) inform policy makers of the guidelines.

## Methods

### Data Sources

A systematic search was conducted in PubMed. We searched all sources from their inception up to January 25, 2021. The search used the following keywords: neuropathic pain, neuralgia, neurodynia, and exercise. The details of the search strategy for the PubMed database are provided in the Supplementary Material.

### Inclusion Criteria

#### Types of Studies

Systematic reviews, meta-analyses, and randomized controlled trials (RCTs) in peer-reviewed journals were included. We excluded retrospective studies, case–control studies, meeting abstracts, conference presentations, book reviews, news items, and corrections. Studies, including Systematic reviews, meta-analyses, and RCTs, with higher levels of evidence were prioritized but lower-quality studies were also evaluated. The language was limited to English.

#### Types of Participants

We included studies that assessed patients suffering from NP caused by spinal cord injury, stroke, multiple sclerosis, Parkinson's disease, cervical radiculopathy, sciatica, diabetic neuropathy, chemotherapy-induced peripheral neuropathy, HIV/AIDS, and surgery (2).

#### Types of Interventions

We only considered studies that involved exercise, such as muscle stretching, strengthening/resistance exercise, aerobic exercise, motor control/stabilization training and mind-body exercise (Tai Chi, yoga, and Pilates). Furthermore, the intervention groups should be able to show the effect of exercise through at least one group. For example, at least one intervention group received exercise only; or one intervention group received exercise combined with usual therapy while another intervention group received usual therapy.

#### Types of Outcome Measures

Outcome measures must include but are not limited to pain intensity. Other outcomes, such as muscle strength, motor functions, and balance, were also considered.

### Study Selection

Two reviewers independently screened the titles, abstracts, and full contents of the proper studies according to the same inclusion criteria. We excluded studies that did not fulfill the inclusion criteria. Any disagreements were resolved by a discussion and a third reviewer was consulted if a disagreement persisted.

### Levels of Evidence

Individual clinical research studies were evaluated in accordance with the criteria adapted from the Oxford Center for Evidence-Based Medicine 2011 Levels of Evidence (CEBM) (available at http://www.cebm.net/index.aspx?o=5653). Two reviewers independently assessed the levels of evidence for each clinical study using an appraisal tool. The abbreviated version of the levels of evidence id shown in Table 1 (18). Additionally, the level of evidence for the recommendation for each disease was determined by the lowest level of evidence from related researched studies.

**Table 1:** Oxford center for evidence based medicine, level of evidence.

**Level**	**Intervention**
I	Evidence obtained from systematic reviews, high-quality diagnostic studies, prospective studies, or randomized controlled trials
II	Evidence obtained from systematic reviews, lesser-quality diagnostic studies, prospective studies, or randomized controlled trials, such as weaker diagnostic criteria and reference standards, improper randomization, no blinding, <80% follow-up
III	Retrospective studies or case-control studies
IV	Case series
V	Expert opinion

### Grades of Recommendation

According to the established clinical guidelines by the American Physical Therapy Association (18–20), the recommendation was graded based on strength of evidence. The authors considered the benefits, side effects of physical therapies, and the strengths and limitations of the evidence body to develop the recommendations. The grades of recommendation are shown in Table 2.

**Table 2:** Grades of recommendation.

**Grades of recommendation**		**Strength of evidence**
A	Strong evidence	A preponderance of level I and/or level II studies support the recommendation. This must include at least 1 level I study
B	Moderate evidence	A single high-quality randomized controlled trial or a preponderance of level II studies support the recommendation
C	Weak evidence	A single level II study or a preponderance of level III and IV studies, including statements of consensus by content experts, support the recommendation
D	Conflicting evidence	Higher-quality studies conducted on this topic disagree with respect to their conclusions. The recommendation is based on these conflicting studies
E	Theoretical/foundational evidence	A preponderance of evidence from animal or cadaver studies, from conceptual models/principles, or from basic science/bench research support this conclusion
F	Expert opinion	Best practice based on the clinical experience of the guideline's development team

### Quality of Evidence

According to Collins et al. (21) and Shea et al. (22), Physiotherapy Evidence Database (PEDro) score (total score/10) and the Assessment of Multiple Systematic Reviews (AMSTAR) score (total score/11) were used to grade the quality of the included RCTs and the methodological quality of the included systematic reviews and meta-analyses, respectively. Two reviewers independently assessed the quality of the included studies through PEDro and AMSTAR. The included studies were graded as low, moderate, or high quality based on the consensus statements (21, 23). Studies with PEDro and AMSTAR scores of ≤ 3, 4–6, and ≥7 were considered to have low, moderate, and high quality, respectively.

### Consensus Process

X-Q.W. and G-E.F. formulated the population, intervention, comparator, and outcome (PICO) research topics and drafted the recommendation statements. During the first round, the 21 experts from the Chinese Association of Rehabilitation Medicine reviewed and commented on the text online using a 5-point scale: 1. strongly agree; 2. agree; 3. no opinion; 4. disagree; 5. strongly disagree (24). A score of 1–2 was determined as “Agreement.” In the second round, the recommendation statements that were regarded as “Disagreement” were discussed further. If 75% agreement could be not gained after discussion, the recommendation statements were further rated in a third round (25). Finally, the grades of recommendation were assigned based on the strength of evidence and a consensus discussion of the results of the Delphi rounds.

## Results

**Figure 1:**
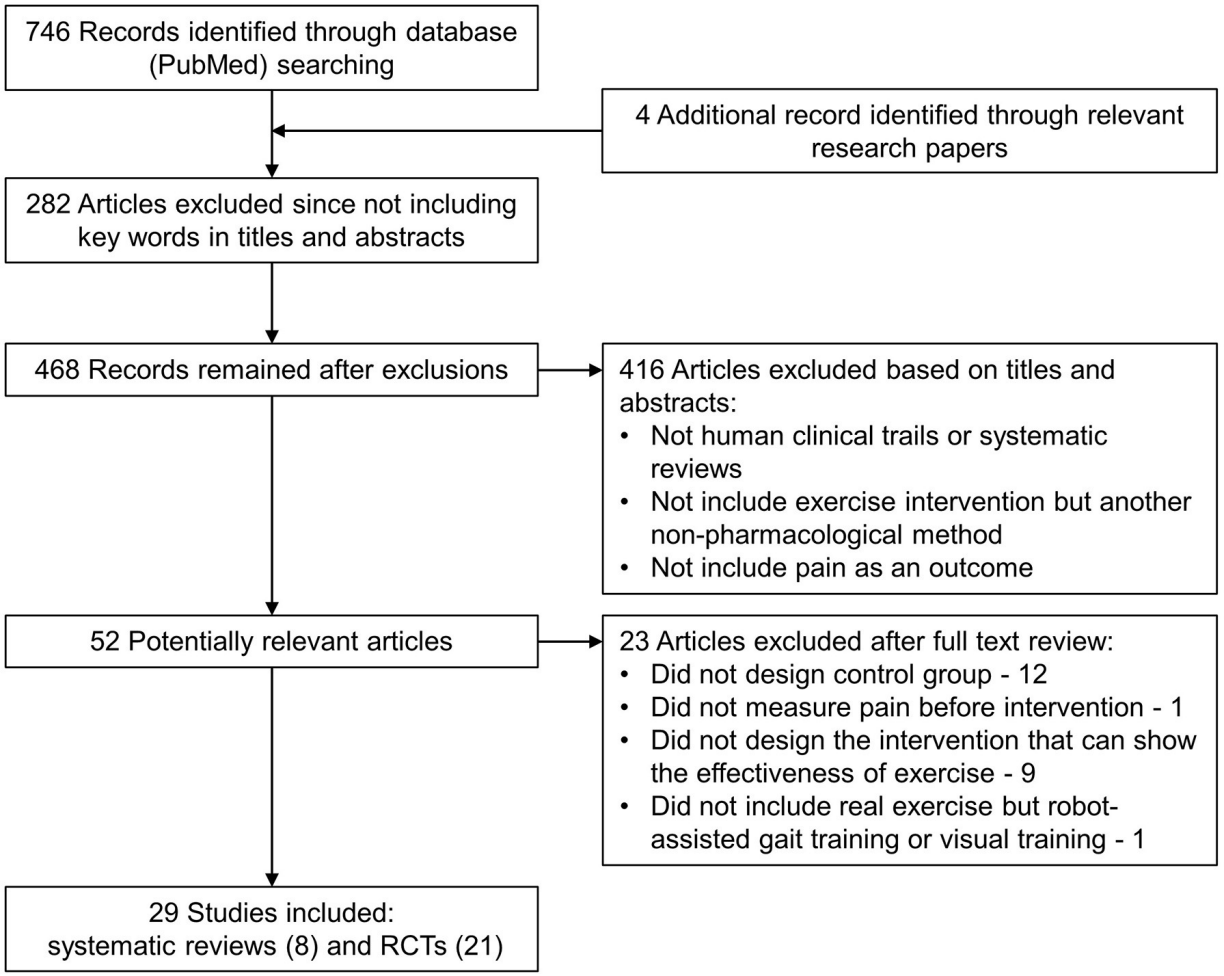
Study flow diagram.

Eight systematic reviews and 21 RCTs met the inclusion criteria after the evaluation of the titles, abstracts, and full contents of the relevant studies (Figure 1). The characteristics and quality of evidence of the included studies are shown in Table 3 (characteristics of RCTs), Table 4 (characteristics of systematic reviews), Table 5 (quality of evidence of RCTs), and Table 6 (quality of evidence of systematic reviews), respectively. Based on PEDro scores, 10 RCTs (47.62%) have high quality, and 11 RCTs (52.38%) have moderate quality. According to AMSTAR scores, six systematic reviews (75%) have high quality, and two systematic reviews (25%) have moderate quality. The summary of the consensus recommendations for exercise as NP treatment is presented in Table 7.

**Table 3:** Characteristics of included RCT studies.

**References**	**Participants**		**Intervention protocol**		**Outcome assessment**	**Outcome measures**	**Results of pain**	**Safety**
	**Population** **Sample size** **Study design**	**Age (years)**	**Intervention group 1**	**Intervention group 2 or control group**				
Labruyère and van Hedel (29)	Patients with incomplete spinal cord injury *n* = 9 2 groups	•59 ± 11 •Group 1 (*n* = 5) •Group 2 (*n* = 4)	•Robot-assisted gait training (45 min per session; 16 sessions within 4 weeks) •Strength training (45 min per session; 16 sessions within the following 4 weeks)	•The same interventions in reversed order	•After the interventions •At follow-up 6 months	•Pain (VAS) •10-m Walk Test •Balance	Robot-assisted gait training and strength training reduced pain intensity.	No adverse events
Costantino et al. (31)	Patients with chronic post-stroke *n* = 32 2 groups	•Group 1 (*n* = 17): 62.59 ± 15.39 •Group 2 (*n* = 15): 60.47 ± 16.06	•Local muscle vibration during voluntary isometric contraction •12 sessions; 3 times per week over 4 weeks	•Voluntary isometric contraction (12 sessions; 3 times per week over 4 weeks)	•After the 4-week interventions	•Pain (VNRS) •Grip strength	Voluntary isometric contraction reduced pain intensity.	NA
Jeon et al. (32)	Patients with post-stroke hemiparesis *n* = 12 2 groups	•Group 1 (*n* = 6): 58.0 ± 13.6 •Group 2 (*n* = 6): 50.5 ± 8.9	•Monkey Chair and Band exercise (joint motion, strengthening training, and relaxation) •30 mins per session; 3 times per week;12 weeks	•No intervention	•At 4 weeks •At 8 weeks •At 12 weeks	•Pain (VAS) •ROM •MMAS	Joint motion and strengthening training reduced pain intensity.	NA
Wei et al. (33)	Patients with hemiplegic shoulder pain *n* = 40 2 groups	•Group 1 (*n* = 20): 63.85 ± 11.07 •Group 2 (*n* = 20): 65.55 ± 13.30	•Acupuncture combined with neuromuscular joint facilitation (NJF) •Once a day; 6 times a week for 3 weeks	•Acupuncture alone •Once a day; 6 times a week for 3 weeks	•After 3-week intervention	•Pain (VAS) •Fugl-Meyer assessment •Passive ROM	NJF training reduced pain intensity.	NA
Horsley et al. (34)	Patients after stroke *n* = 50 2 groups	•Group 1 (*n* = 25): 65.9 ± 12.7 •Group 2 (*n* = 25): 68.5 ± 13.0	•Active, intensive, repetitive upper limb training using the SMART Arm device (1 h a day; 5 days a week for 5 weeks) •Upper limb therapy (5 days a week for the same 5 weeks)	•Usual upper limb therapy (5 days a week for 5 weeks)	•After 5-week interventions •At 7-week follow-up	•Pain (VAS)	No significant effects of upper limb training by SMART Arm device on pain intensity.	NA
Pilutti et al. (38)	Patients with Multiple Sclerosis *n* = 8/11 2 groups	•Group 1 (*n* = 4): 57.3 ± 6.0 •Group 2 (*n* = 4): 48.5 ± 7.7	•Functional electrical stimulation (FES) cycling exercise •Same cadence (50 rpm); 3 weekly sessions for 24 weeks	•Passive leg cycling •3 weekly sessions for 24 weeks	•After 24-week intervention	•Pain (McGill Pain Questionnaire) •Cognitive processing speed •Symptoms of fatigue	The FES cycling exercise reduced pain intensity.	Six mild adverse events
Grubić Kezele et al. (39)	Patients with Multiple Sclerosis *n* = 19 2 groups	•Group 1 (*n* = 10): 53.9 ± 10.7 •Group 2 (*n* = 9): 48.2 ± 9.3	•Combined upper limb and breathing exercise (60 mins per session; 2 sessions a week for 4 weeks) •Independent home exercise (20 mins per session; 3 sessions per week for the same 4 weeks) •On-going physical therapy	•On-going physical therapy without exercise	•After 4-week intervention	•Pain (Short-form 36) •Fatigue •Quality of life	The combined upper limb, breathing and home exercise reduced pain intensity.	NA
Hasanpour-Dehkordi et al. (40)	Patients with Multiple Sclerosis *n* = 60 2 groups	•30.0 •Group 1 (*n* = 30) •Group 2 (*n* = 30)	•Yoga exercises •60–70 mins 3 sessions a week for 12 weeks	•No exercise	•After 12-week intervention	•Pain (Bayer numerical scale) •Fatigue severity	Yoga exercise reduced pain symptoms.	NA
Young et al. (41)	Patients with Multiple Sclerosis *n* = 81 3 groups	•Group 1 (*n* = 27): 49.67 ± 9.40 •Group 2 (*n* = 26): 48.35 ± 9.95 •Group 3 (*n* = 28): 47.29 ± 10.33	Group 1: movement-to-music •Strength, cardiorespiratory endurance, and balance •60 mins per session; 3 sessions per week for 12 weeks Group 2: adapted yoga •60 mins per session; 3 sessions per week for 12 weeks	•Waitlist control (biweekly newsletters *via* mail)	•After 12-week intervention	•Pain (PROMIS) •Timed Up and Go •6-min walk test	No significant effect of movement-to-music or adapted yoga on pain intensity.	One muscle strain in movement-to-music group
Pérez de la Cruz (43)	Patients with Parkinson's disease *n* = 30 2 groups	•Group 1 (*n* = 15): 66.80 ± 5.867 •Group 2 (*n* = 15): 67.53 ± 9.89	•Aquatic Tai Chi •45 mins per time; 2 times per 10 weeks	•Therapy on dry land (strength training and aerobic exercises) •45 mins per time; 2 times per 10 weeks	•After 10-week intervention •At 1-month follow-up	•Pain (VAS) •Balance •Test get up and go •Five times test	Both aquatic Tai Chi and strength training and aerobic exercises reduced pain intensity.	NA
Diab and Moustafa (44)	Patients with unilateral lower cervical spondylotic radiculopathy *n* = 96 2 groups	•Group 1 (*n* = 48): 46.3 ± 2.05 •Group 2 (*n* = 48): 45.9 ± 2.1	•Posture corrective exercise program (strengthening and stretching exercise; 4 times per week for 10 weeks) •Ultrasound and infrared radiation (20 mins per time; 3 times per week for 10 weeks)	•Ultrasound and infrared radiation •20 mins per time; 3 times per week for 10 weeks	•After 10-week intervention •At 6-month follow-up	•Pain (VAS) •Somatosensory evoked potentials •Craniovertebral angle	Strengthening and stretching exercise significantly reduced pain intensity.	NA
Halvorsen et al. (45)	Patients with cervical radiculopathy *n* = 50/75 2 groups	•Group 1 (*n* = 27): 47 ± 10.9 •Group 2 (*n* = 23): 49 ± 9.4	•Neck-specific training with a cognitive behavioral approach •Prescribed physical activity •3 times a week for 14 weeks	•Prescribed self-mediated physical activity	•After 14-week intervention •At 12-month follow-up	•Pain (VAS) •Neck endurance test	Neck-specific training and physical activity reduced neck pain intensity.	No adverse events
Fritz et al. (46)	Patients with neck pain and signs of radiculopathy *n* = 86 3 groups	•Group 1 (*n* = 31): 48.1 ± 10.0 •Group 2 (*n* = 27): 47.6 ± 10.9 •Group 3 (*n* = 28): 44.9 ± 11.3	Group 1: exercise with mechanical traction •Exercise (scapula and cervical strengthening) •Mechanical cervical traction during treatment •30–45 mins per session; 10 sessions over a 4-week treatment Group 2: exercise with over-door traction •Exercise (scapula and cervical strengthening) •Traction using a Chattanooga Overdoor Traction Device during treatment •30–45 mins per session; 10 sessions over a 4-week treatment	Group 3: exercise •Scapular and cervical strengthening •30–45 mins per session; 10 sessions over a 4-week treatment	•After 4-week intervention •At 6-month follow-up •At 12-month follow-up	•Pain (pain catastrophizing scale) •Neck disability	Scapula and cervical strengthening with or without traction reduced pain intensity.	5.6% severe adverse events
Albert and Manniche (48)	Patients with radicular pain below the knee *n* = 181 2 groups	•Group 1 (*n* = 95): 46 (38–52) •Group 2 (*n* = 96): 44 (37–51)	•Symptom-guided exercises (stabilizing and dynamic exercises) •Information •Advice to stay active •4–8 times for 8 weeks	•Sham exercises (not back related) •Information •Advice to stay active •4–8 times for 8 weeks	•At 8-week intervention •At 1-year follow-up	•Pain (VAS) •Global improvement •Functional status	Symptom-guided exercise reduced leg pain.	NA
Cox et al. (50)	Patients with type 2 diabetes *n* = 32 3 groups	•Group 1 (*n* = 10): 57.8 ± 6.9 •Group 2 (*n* = 10): 58.7 ± 9.2 •Group 3 (*n* = 12): 59.5 ± 11.1	Group 1: supervised combined aerobic and resistance moderate-intensity continuous training (C-MICT) •52.5 mins per time; 4 times per week for 8 weeks Group 2: supervised combined high-intensity interval training (C-HIIT) •26 mins per time; 3 times per week for 8 weeks	•Group 3: usual care •8 weeks	•After 8-week intervention	•Pain (VAS) •Neuropathy symptom	C-HIIT and C-MICT exercise groups showed reduction in pain intensity but not neuropathic symptoms.	Nineteen mild adverse events in C-HIIT group; 17 in C-MICT group.
Win et al. (51)	Patients with diabetic peripheral neuropathy *n* = 75/104 2 groups	•Group 1 (*n* = 32): 55.38 ± 9.54 •Group 2 (*n* = 43): 55.72 ± 10.55	•Simple hand, finger, and foot exercises •Three times a week for 8 weeks	•Usual care •Diabetic foot care education	•After 8-week intervention •At 16-week follow-up	•Pain (VAS) •Activities of daily living	Simple hand, finger, and foot exercises reduced pain intensity.	No adverse event
Hwang et al. (54)	Female patients before radiotherapy after various operations *n* = 37/40 2 groups	•Group 1 (*n* = 17): 46.3 ± 7.5 •Group 2 (*n* = 20): 46.3 ± 9.5	•Supervised moderate-intensity exercise (stretching, aerobic and strengthening exercise) •50 mins per time; 3 times per week for 5 weeks	•Self-shoulder stretching education and advice to normal activities	•After 5-week intervention	•Pain (VAS) •QOL •Shoulder range of motion	Supervised moderate-intensity exercise reduced pain intensity.	No significant adverse events
Dhawan et al. (55)	Patients with neck pain and signs of radiculopathy *n* = 45 2 groups	•Group 1 (*n* = 22): 50.5 ± 7.9 •Group 2 (*n* = 23): 52.5 ± 6.6	•Home-based muscle strengthening •Balancing exercise •30 mins daily for 10 weeks	•Usual care	•After 10-week intervention	•Pain (Leeds Assessment of Neuropathic Symptoms and Signs) •QOL	Muscle strengthening and balancing exercise significantly reduced neuropathic pain.	No adverse effects
Maharaj and Yakasai (57)	Patients with HIV-induced distal symmetrical polyneuropathy *n* = 136 3 groups	•Group 1 (*n* = 45): 38.29 ± 8.06 •Group 2 (*n* = 44): 35.98 ± 8.53 •Group 3 (*n* = 47): 36.13 ± 8.10	Group 1: Aerobic exercise •30 mins per time; 3 times per week for 12 weeks Group 2: progressive resisted exercise •30 mins per time; 3 times per week for 12 weeks	Group 3: control group •Attended HIV talks, video presentations, and counseling	•After 6-week intervention •After 12-week intervention	•Pain (VNRS)	Aerobic exercise and progressive resisted exercise significantly reduced pain intensity.	No adverse effects
Tumusiime et al. (58)	Patients with HIV-associated peripheral neuropathy *n* = 120 2 groups	•Group 1 (*n* = 60): 41.2 ± 7.8 •Group 2 (*n* = 60): 40.4 ± 7.7	•Physiotherapy-led aerobic exercises (stretching, strengthening and balance exercises) •Routine Health Care •60 mins per each session; 3 times a week for 12 weeks	•Routine Health Care	•After 12-week intervention •At 12-week follow-up	•Pain (VNRS)	Physiotherapist-led aerobic exercises reduced neuropathic pain.	NA
Ammitzbøll et al. (62)	Female patients after breast cancer surgery *n* = 158 2 groups	•Group 1 (*n* = 82): 53 ± 10 •Group 2 (*n* = 76): 53 ± 10	•Supervised and self-administered, progressive resistance training intervention initiated 3 weeks after surgery •The first 20-week physiotherapist-led exercise (weekly once) •The following 30-week self-administered exercise	•Usual care (information concerning post-operative care and mobility exercises)	•After 20-week intervention •After 12-month intervention	•Pain (VNRS)	Supervised and self-administered, progressive resistance training reduced pain intensity.	NA

*Age were showed as mean ± SD or median (25–75% Interquartile Range). Simple size was shown as “the number of finial analysis”/“the number of included patients.”*

*VAS, visual analog scale; VNRS, verbal numerical rating scale; ROM, range of motion; MMAS, modified motor assessment scale; PROMIS, patient-reported outcomes measurement information system; NA, not available*.

**Table 4:** Characteristics of included systematic reviews.

**References**	**Participants**	**Study and sample size**	**Intervention**	**Control**	**Results of pain**
Boldt et al. (27) and Harvey et al. (15)	Patients with spinal cord injury	3 RCT, *n* = 149	Exercise (stretching and strengthening exercises)	Control (no treatment or 1-h educational video control)	Exercise decreased pain intensity.
Gómara-Toldrà (28)	Patients with spinal cord injury	5 trials, *n* = 81	Exercise (treadmill training, strengthening, and stretching exercises)	NA	Exercise decreased pain intensity.
Demaneuf et al. (36)	Patients with multiple sclerosis	10 RCT, *n* = 389	Exercise (aerobic, resistance, and the combination exercises)	Passive control groups (waiting list or normal treatment)	Exercise decreased pain intensity.
Fernandez et al. (47)	Patients With Sciatica	5 RCT, *n* = 604	Exercise (Stabilization exercises, hydrotherapy and isometric exercises.)	Advice to stay active	Exercise decreased pain intensity.
Tough et al. (53)	Patients with cancer	1 trial, *n* = 81	Exergaming (Breakout 3D, Card Island and other exergaming)	NA	Exercise decreased pain intensity.
McNeely et al. (60) and De Groef et al. (61)	Female patients following breast cancer surgery	1 trial, *n* = 30	Exercise (exercise for arm/shoulder, posture correction, coordination, and strengthening exercises)	Leaflet with advice and exercises for arm and shoulder	Exercise decreased pain intensity.

*RCT, randomized controlled trial; NA, not available*.

**Table 5:** Physiotherapy evidence database scores of included RCT studies.

**References**	**Random allocation**	**Concealed allocation**	**Baseline comparability**	**Blind subjects**	**Blind therapists**	**Blind assessors**	**Adequate follow-up**	**Intention-to-treat analysis**	**Between-group comparisons**	**Point estimates and variability**	**Total (0–10 Scale)**	**Quality**
Labruyère and van Hedel (29)	Yes	No	No	No	No	Yes	Yes	Yes	Yes	Yes	6	Moderate
Costantino et al. (31)	Yes	No	Yes	No	No	Yes	Yes	No	Yes	Yes	6	Moderate
Jeon et al. (32)	Yes	No	Yes	No	No	No	Yes	No	Yes	Yes	5	Moderate
Wei et al. (33)	Yes	No	Yes	No	No	No	No	No	Yes	Yes	4	Moderate
Horsley et al. (34)	Yes	Yes	Yes	No	No	Yes	Yes	Yes	Yes	Yes	8	High
Pilutti et al. (38)	Yes	No	Yes	No	No	Yes	No	No	Yes	Yes	5	Moderate
Grubić Kezele et al. (39)	Yes	No	Yes	No	No	Yes	Yes	Yes	Yes	Yes	7	High
Hasanpour-Dehkordi et al. (40)	Yes	No	Yes	No	No	No	Yes	No	Yes	Yes	5	Moderate
Young et al. (41)	Yes	Yes	Yes	No	No	Yes	No	Yes	Yes	Yes	7	High
Pérez de la Cruz (43)	Yes	No	Yes	No	No	Yes	Yes	Yes	Yes	Yes	7	High
Diab and Moustafa (44)	Yes	Yes	Yes	No	No	No	Yes	Yes	Yes	Yes	7	High
Halvorsen et al. (45)	Yes	Yes	Yes	No	No	No	No	No	Yes	Yes	5	Moderate
Fritz et al. (46)	Yes	Yes	Yes	No	No	Yes	Yes	Yes	Yes	Yes	8	High
Albert and Manniche (48)	Yes	No	Yes	No	No	Yes	Yes	Yes	Yes	Yes	7	High
Cox et al. (50)	Yes	Yes	Yes	No	No	No	No	Yes	Yes	Yes	6	Moderate
Win et al. (51)	Yes	No	Yes	No	No	No	No	No	Yes	Yes	4	Moderate
Hwang et al. (54)	Yes	No	Yes	No	No	No	Yes	No	Yes	Yes	5	Moderate
Dhawan et al. (55)	Yes	Yes	Yes	No	No	No	Yes	Yes	Yes	Yes	7	High
Maharaj and Yakasai (57)	Yes	No	Yes	No	No	Yes	Yes	No	Yes	Yes	6	Moderate
Tumusiime et al. (58)	Yes	Yes	Yes	No	No	Yes	Yes	Yes	Yes	Yes	8	High
Ammitzbøll et al. (62)	Yes	No	Yes	No	No	Yes	Yes	Yes	Yes	Yes	7	High

*High quality: total score ≥7; moderate quality: total score 4–6; low quality: total score ≤ 3*.

**Table 6:** Quality ratings of included systematic reviews evaluated using AMSTAR.

**References**	**Priori design**	**Duplicate study selection and data extraction**	**Comprehensive literature search**	**Search for gray literature**	**List of studies included and excluded provided**	**Characteristics of included studies provided**	**Scientific quality assessed**	**Scientific quality used to formulate conclusions**	**Methods to combine study findings appropriate**	**Publication bias assessed**	**Conflict of interest**	**Total (0–11 Scale)**	**Quality**
Harvey et al. (15)	Yes	Yes	Yes	No	No	Yes	Yes	Yes	Yes	CA	Yes	8	High
Boldt et al. (27)	Yes	Yes	Yes	Yes	Yes	Yes	Yes	Yes	Yes	Yes	Yes	11	High
Gómara-Toldrà et al. (28)	Yes	Yes	Yes	CA	No	Yes	Yes	Yes	No	No	No	6	Moderate
Demaneuf et al. (36)	Yes	Yes	Yes	CA	No	Yes	Yes	Yes	Yes	Yes	No	8	High
Fernandez et al. (47)	Yes	Yes	Yes	No	No	Yes	Yes	Yes	Yes	NA	No	7	High
Tough (53)	Yes	Yes	Yes	CA	No	Yes	Yes	Yes	No	Yes	Yes	8	High
McNeely (60)	Yes	Yes	Yes	Yes	Yes	Yes	Yes	Yes	Yes	CA	Yes	10	High
De Groef et al. (61)	Yes	Yes	Yes	CA	No	Yes	Yes	Yes	No	No	No	6	Moderate

*High quality: total score ≥7; moderate quality: total score 4–6; low quality: total score ≤ 3.*

*CA, cannot answer; NA, not applicable*.

**Table 7:** Recommendation summary of exercise for neuropathic pain management.

**Neuropathic pain**	**Recommendations**	**Level of evidence**	**Grades of recommendation**	**Consensus**
Spinal cord injury	Yes	II	A	100% Yes (21 voters)
Post-stroke pain	Yes	II	C	95% Yes (21 voters)
Multiple sclerosis	Yes	II	B	95% Yes (21 voters)
Parkinson's disease	Yes	II	C	100% Yes (21 voters)
Cervical radiculopathy	Yes	II	B	100% Yes (21 voters)
Sciatica	Yes	I	A	100% Yes (21 voters)
Diabetic neuropathy	Yes	II	B	95% Yes (21 voters)
Chemotherapy-induced peripheral neuropathy	Yes	II	B	95% Yes (21 voters)
Neuropathy due to HIV/AIDS	Yes	II	B	100% Yes (21 voters)
After surgery for breast cancer	Yes	I	A	86% Yes (21 voters)

### Consensus Recommendations for Pain Associated With Spinal Cord Injury

Chronic pain is a common and serious symptom in patients with spinal cord injury with a high prevalence at around 73% in Denmark (26). About 30% of patients considered the pain as a severe health problem that influences their physical and mental functions and daily lives. Three systematic reviews reported that exercise is effective in relieving pain in patients with spinal cord injury (15, 27, 28). Two systematic reviews (15, 27), which involved the same three RCTs (*n* = 149 patients with spinal cord injury) illustrated that both short–term and long–term stretching and strengthening exercises can decrease chronic shoulder pain through the 36-Item Short Form Survey for pain experience [weight mean difference (WMD) = −1.9, 95% CI = −3.4 to −0.4, *P* = 0.01] and pain visual analog scale (WMD = −2.8, 95% CI = −3.77 to −1.83, *P* < 0.00001) compared with no treatment or 1-h educational video control (Level of evidence I). Furthermore, a randomized cross-over study by Labruyère and van Hedel (29) (*n* = 9) found that strength training (−6.8% ± 2.5%) and robot-assisted gait training (−4.5% ± 2.2%) can relieve pain experience during single training intervention and after 16 sessions in patients with incomplete spinal cord injury. Meanwhile, the immediate pain relief was slight whereas integral effect was substantial, and the difference in pain is in favor of strength training compared with robot-assisted gait training (*P* < 0.01). Additionally, strength training could improve the 10-meter walk test and balance function (Level of evidence II).

#### Expert Recommendation

We recommend using exercise programs, such as stretching and strengthening exercises, as treatment for NP in patients with spinal cord injury (Level of evidence II, A).

### Consensus Recommendations for Post-stroke Pain

Up to 50% of patients with stroke report pain after stroke (30). Post-stroke chronic pain makes motor function, cognition, quality of life, and depression worse. However, post-stroke pain is commonly underestimated by patients and physicians who more focus on hemiplegia and deficient motor functions. One RCT by Costantino et al. (31) including 32 patients with stroke found that the subjects who underwent voluntary isometric muscle contraction in upper extremities with and without vibrations for 4 weeks reported decreased pain (Level of evidence II). According to another RCT (32) on 12 post-stroke patients, the participants who did joint motion and strengthening training *via* a specific exercise tool experienced less pain than the participants without intervention at 4, 8, and 12 weeks (Level of evidence II). Similarly, a RCT (*n* = 40) pointed out that 3-week neuromuscular joint facilitation combined with acupuncture can reduce post-stroke pain more than 3-week pure acupuncture therapy (33) (Level of evidence II). By contrast, another RCT involving 50 patients with stroke observed that a 1-h active, high-intensity, and repetitive training of the upper extremities had no clinically important effects on pain intensity compared with usual upper limb therapy (34) (Level of evidence II). The different findings might be caused by the specific training device, called SMART Arm, or severe physical condition of participants, which is no more than 90° of the affected shoulder flexion.

#### Expert Recommendation

We recommend using strengthening exercise and neuromuscular joint facilitation as a treatment for patients with post-stroke pain (Level of evidence II, C).

### Consensus Recommendations for Pain Associated With Multiple Sclerosis

Multiple sclerosis is characterized by demyelination and axonal loss in the central nervous system accompanied by NP. According to previous studies, 29–86% of patients with multiple sclerosis suffer from NP, leading to depression and low-quality of life (35). One systematic review and two RCTs reported the effectiveness of exercise training in relieving pain in patients with multiple sclerosis. The systematic review and meta-analysis of 10 RCTs involving 389 patients by Demaneuf et al. (36) demonstrated that exercise interventions, including single aerobic exercise, aquatic aerobic exercise, resistance training, and the combination of these interventions, have more positive effects on the pain intensity by patients with multiple sclerosis than passive control groups [standardized mean difference (SMD) = −0.46, 95% CI = −0.92 to 0.00, between-study heterogeneity (*I*^2^) = 77.0%; Level of evidence I]. A RCT (37, 38) showed that 24-week cycling exercise with functional electrical stimulation could help alleviate pain in comparison with passive leg cycling exercise (SMD = −0.67; Level of evidence II). Similarly, another RCT illustrated that 4-week upper extremity, breathing and independent home exercises has a trend toward less pain than physical therapy without exercise (39) (Level of evidence II).

Furthermore, a RCT (*n* = 60) reported that the exercise group with 12-week yoga intervention showed an improvement in pain intensity and physiological indices compared with the group without exercise (40) (Level of evidence II). However, one three-arm RCT (*n* = 81) reported by Young et al. (41) argued that the pain conditions among 12-week movement to music, adapted yoga with a series of stationary poses, and waitlist control have no substantial differences (Level of evidence II). The conflicting results could be explained by different study design, such as participants, control groups and movement positions during yoga.

#### Expert Recommendation

We recommend using aerobic, aquatic aerobic, and resistance training as pain treatment for patients with multiple sclerosis (Level of evidence II, B).

### Consensus Recommendations for Pain Associated With Parkinson's Disease

In addition to dystonia, pain is another serious symptom that impacts the motor function, depression condition, and daily lives of patients with Parkinson's disease (42). A single-blinded RCT (*n* = 30) found that 10-week aquatic Tai Chi training and usual exercise that focuses on gait, balance, and muscle strength can decrease pain intensity in people with Parkinson's disease (43) (Level of evidence II). Moreover, aquatic Tai Chi is more superior than usual exercise in pain reduction and gait and balance condition improvement.

#### Expert Recommendation

We recommend using aquatic Tai Chi, muscle strengthening training, and balance exercise as treatment for pain in patients with Parkinson's disease (Level of evidence II, C).

### Consensus Recommendations for Painful Radiculopathy

#### Cervical Radiculopathy

Cervical radiculopathy is a subgroup of neck pain characterized by pain radiating along the affected arms. The sort and intensity of symptoms, such as NP and muscle weakness, depend on the extent of cervical spinal nerve root compression. Three RCTs suggestion the use of an exercise program as a treatment for subjects with cervical radiculopathy. According to Diab and Moustafa (44), one RCT (*n* = 96) reported that a 10-week physical exercise comprised of neck muscle strengthening and stretching combined with ultrasound and infrared radiation are more effective on pain relief than the combination of ultrasound and infrared radiation in the short term and 6-month follow-up (Level of evidence II). Another RCT, which involved 75 patients with cervical radiculopathy, suggested that 14 weeks of neck-specific training targeting sensory and motor function and 14-week physical activities can reduce NP intensity and increase the endurance of neck flexors in the long term (45) (Level of evidence II). Furthermore, a RCT (*n* = 86) reported by Fritz et al. (46) suggested that although exercise that aimed to strengthen the scapula and cervical muscles are helpful to alleviate neck and arm pain, the combination of exercise and mechanical traction has a greater advantage in pain relief and function improvement than single exercise at 4-week, 6-month, and 12-month time points (Level of evidence II).

##### Expert Recommendation

We recommend using exercise training that targets neck muscle strength and stretch as treatment or complementary therapy for NP associated with cervical radiculopathy (Level of evidence II, B).

#### Sciatica

Sciatica is a defined as a subgroup of low back pain with a specific symptom, that is, radicular leg pain radiating along the distribution of the sciatic nerve (14). Although the prevalence of sciatica is much lower than low back pain, the affected region and prognosis are normally more severe; therefore, sciatica contributes a high degree of hopelessness and depression. A systematic review and meta-analysis (5 RCTs, *n* = 604) pointed out that an exercise program comprised of static and dynamic stabilizing exercises, hydrotherapy, and isometric exercises that target the trunk and lower extremity muscles is beneficial to leg pain reduction (WMD = 11.43, 95% CI = 0.71–22.16) but not disability (WMD = 1.45, 95% CI = −2.86–5.76) in the short term compared with advice to stay active among patients suffering from sciatica (47) (Level of evidence I). According to a single-blinded RCT that involved 181 patients with severe sciatica by Albert and Manniche (48), symptom-guided exercises, such as postural instructions, stabilizing exercises for deep muscles, and dynamic exercises for surface muscles in the trunk region, had a trend to a larger reduction of leg pain than the sham exercise group that performed low-intensity and no back-related training (Level of evidence I).

##### Expert Recommendation

We recommend using motor control, aquatic stabilizing movements, and isometric exercises that target the trunk and lower extremity muscles as an adjunct treatment for pain in patients with sciatica (Level of evidence I, A).

### Consensus Recommendations for Painful Polyneuropathy

#### Diabetic Neuropathy

Diabetic peripheral neuropathy marked by pain and sensory and mobility loss, is a common and often disabling complication of diabetes mellitus (49). Diabetic neuropathy has been considered a serious problem because its treatments are likely ineffective. Two clinical trials investigated the effectiveness of exercise training on pain in diabetic neuropathy. One three-arm RCT divided 32 inactive patients with type 2 diabetes into three treatment groups: usual care, the combination of aerobic exercise and continuous moderate-intensity resistance training, and the combination of aerobic exercise and high-intensity interval training (50). The findings suggested that 8-week moderate-intensity and high-intensity exercise interventions are more beneficial in decreasing pain intensity but not neuropathic symptoms compared with single usual care lasting for 8 weeks (Level of evidence II). Particularly, the combination of aerobic exercise and high-intensity interval training significantly alleviated pain intensity. Based on another RCT (*n* = 104) comparing an 8-week simple hand, finger, and foot exercise with health education and control group with health education by Win et al. (51), both groups appeared decreased pain and the exercise intervention could relieve more pain than the control group in the short term and at 16-month follow-up (Level of evidence II).

##### Expert Recommendation

We recommend using general exercise focusing on distal extremities, or the combination of aerobic and moderate-intensity or high-intensity exercises, as a treatment for pain in patients with diabetes (Level of evidence II, B).

### Chemotherapy-Induced Peripheral Neuropathy

Chemotherapy-induced peripheral neuropathy, a common side effect of cancer treatment with a prevalence of 30–80%, is a small-fiber sensory neuropathy in the hands or feet (17). The typical symptoms are shooting pain, stabbing pain, or burning pain, which progressively becomes worse with chemotherapy (52). A systematic review by Tough et al. (53) mentioned that one pre–post clinical trial found a slight reduction in pain intensity and improvement of balance, motor functions, and depression status with higher adherence rates and enjoyment after 8 weeks of progressive exergaming program, which is a combination of exercise and games (Level of evidence II). Similarly, in a RCT study (*n* = 40), Hwang et al. (54) found that a 30-min exercise program that includes stretching and aerobic training could reduce pain and improve motor functions in patients after radiotherapy more than self-stretching training after 5 weeks of intervention (Level of evidence II). According to one RCT involving 45 patients with cancer reported by Dhawan et al. (55), a 10-week muscle strength and balance training has more positive effects on decreasing the NP intensity (*P* < 0.0001) and increasing quality of life (*P* = 0.0002) in patients with cancer who suffer from chemotherapy-induced peripheral neuropathy compared with usual care (Level of evidence II).

#### Expert Recommendation

We recommend muscle strengthening and balance training as treatment and exergaming as adjunct therapy for chemotherapy-induced NP (Level of evidence II, B).

## Neuropathy Due to HIV/AIDS

Up to 90% of patients with HIV/AIDS complain about pain due to various reasons, including viral infection of the peripheral or central nervous system and side effects of anti-retroviral therapy (56). A three-arm RCT compared 12-week aerobic exercise (cycling), progressive resistance exercise focused on muscles in the lower extremities, and no exercise control among 136 patients with HIV (57). The findings suggested that aerobic and progressive resistance exercise are helpful and safe in the treatment of NP compared with no exercise at 6- and 12-week points (Level of evidence II). Moreover, one high-quality RCT, which involved 120 patients with HIV who underwent anti-retroviral treatment, supported that supervised aerobic exercise, including isometric, balance, and breath training, could alleviate NP more than non-exercise control after 12 weeks of intervention and at the 12 weeks of follow-up (58) (Level of evidence II).

### Expert Recommendation

We recommend aerobic and progressive resistance training as an adjunct treatment for NP in people with HIV/AIDS (Level of evidence II, B).

## Consensus Recommendations for NP Following Surgery

Chronic postsurgical pain is multifactorial and affects up to 50% of patients who underwent operation. Surgeries operated in the thorax, breast, and hernia regions and those that easily produce nerve injury have a high risk of postsurgical NP (59). According to a systematic review by McNeely et al. (60), exercise intervention is more effective in improving pain intensity than usual care despite no significant difference and has no adverse effects for post-operation patients with breast cancer after 3 weeks of intervention or in the 6-month follow-up (Level of evidence I). Additionally, one systematic review reported that posture correction and strengthening exercises are more beneficial to alleviate the post-operation pain and improve the motor functions in patients undergoing breast cancer surgery compared with education only or no intervention (61) (Level of evidence I). One RCT that consists of 158 female patients with breast cancer who underwent axillary lymph node dissection showed that a 12-month self-administered progressive resistance exercise program focusing on the whole body could alleviate NP more than usual care (62) (Level of evidence I).

### Expert Recommendation

We recommend using muscle strengthening and posture correction as treatment for NP after operation for breast cancer (Level of evidence I, A).

## Discussion

The study aimed to review the effectiveness and efficacy of exercise on diseases with neuropathic pain through evidence, thereby producing evidence-based exercise recommendations for NP and informing medical staff and policymaker about the formulation of exercise prescription. A total of eight systematic reviews and 21 RCTs were included, which involved various exercise, such as strengthening, stretching, aquatic aerobic, balance trainings. Finally, 10 recommendations for NP caused by different disorders, including spinal cord injury, stroke, multiple sclerosis, Parkinson's disease, cervical radiculopathy, sciatica, diabetic neuropathy, chemotherapy-induced peripheral neuropathy, HIV/AIDS, and surgery, were described. Various exercise programs may have some benefits in improving pain and functions and proper exercise can be used as an effective alternative treatment or complementary therapy for different disorders with NP.

This paper was the first expert consensus to report exercise recommendations for different diseases with NP, including spinal cord injury, stroke, multiple sclerosis, Parkinson's disease, cervical radiculopathy, sciatica, diabetic neuropathy, chemotherapy-induced peripheral neuropathy, surgery, and HIV/AIDS. We searched studies published before January 2021. Then, the grades of recommendations were based on the strength of evidence and a consensus discussion of the results of the Delphi rounds. In addition, we used PEDro and AMSTAR to assess the quality of the included RCTs and systematic reviews. Finally, all studies that met the inclusion criteria and were deemed to have levels of evidence of I and II were included in this expert consensus.

Some limitations have to be considered in this study. First, the recommendations were made through qualitative analysis in this consensus whereas more specific and rigorous clinical recommendations that include the types, intensity, and frequency of exercise should be decided by quantitative analysis. Second, different from most guidelines that used Grading of Recommendations, Assessment, Development and Evaluations to make clinical practice recommendations (63, 64), we assessed the levels of evidence through Oxford CEBM and evaluated the grades of recommendation according to the methods established by the American Physical Therapy Association. Moreover, most of the RCTs (47.62%) and systematic reviews (75%) have high quality based on PEDro and AMSTAR scores, respectively. Nevertheless, some outcomes of the meta-analyses had considerable heterogeneity, thereby providing relatively inferior evidences. Furthermore, because studies on the various exercise for NP are limited, we did not summarize the recommendations according to different types of exercise, such as aerobic exercise or progressive resistance training, or provide detailed information about intensity, time, or frequency of exercise prescription. Finally, we did not adequately describe the effect of exercise on other aspects among patients with difference diseases since the NP intensity was the focus in the study.

## Conclusion

Exercise can be considered as a feasible, and effective alternative treatment or complementary therapy for most patients with NP caused by different diseases. An updated consensus statement will be required if adequate new studies will be available in the future. This consensus statement will require regular updates after 5–10 years to guarantee that treatments and recommendations continue to be supported by the latest evidence. More high-quality randomized controlled trails are required to provide more superior evidence in the future. Exercise with various types, intensities, and frequencies; patient preference; and facility conditions should be considered as well in further studies.

## Data Availability Statement

The original contributions presented in the study are included in the article/Supplementary Material, further inquiries can be directed to the corresponding author/s.

## Author Contributions

G-EF and X-QW: conceptualization and supervision. Y-HZ, Y-CX, GP, LH, Y-ZK, Y-LW, J-BG, SB, T-SL, L-JA, C-HW, Y-LB, LF, CM, L-RL, HL, YZ, Z-JZ, C-LL, G-EF, and X-QW: methodology and visualization. Y-CX, CP, LH, Y-ZK, Y-LW, J-BG, SB, T-SL, L-JA, C-HW, Y-LB, LF, CM, L-RL, HL, YZ, Z-JZ, C-LL, G-EF, and X-QW: validation. Y-HZ and H-YH: writing—original draft preparation. Y-HZ and X-QW: writing—review and editing. All authors have read and agreed to the published version of the manuscript.

## Funding

This work was supported by the Science and Technology Commission of Shanghai Municipality (21S31902400) and the Shanghai Key Lab of Human Performance (Shanghai University of Sport) (11DZ2261100).

## Conflict of Interest

The authors declare that the research was conducted in the absence of any commercial or financial relationships that could be construed as a potential conflict of interest.

## Publisher's Note

All claims expressed in this article are solely those of the authors and do not necessarily represent those of their affiliated organizations, or those of the publisher, the editors and the reviewers. Any product that may be evaluated in this article, or claim that may be made by its manufacturer, is not guaranteed or endorsed by the publisher.

## Supplementary Material

Supplementary Material 1 The details of the search strategy for the PubMed database.

## References

[1] Colloca L Ludman T Bouhassira D Baron R Dickenson A Yarnitsky D . Neuropathic pain. Nat Rev Dis Primers. (2017) 3:17002. 10.1038/nrdp.2017.2 28205574 PMC5371025

[2] Finnerup N Kuner R Jensen T. Neuropathic pain: from mechanisms to treatment. Physiol Rev. (2021) 101:259–301. 10.1152/physrev.00045.2019 32584191

[3] Burke D Fullen B Stokes D Lennon O. Neuropathic pain prevalence following spinal cord injury: a systematic review and meta-analysis. Eur J Pain. (2017) 21:29–44. 10.1002/ejp.905 27341614

[4] Bouhassira D Lantéri-Minet M Attal N Laurent B Touboul C. Prevalence of chronic pain with neuropathic characteristics in the general population. Pain. (2008) 136:380–7. 10.1016/j.pain.2007.08.013 17888574

[5] Finnerup N Attal N Haroutounian S McNicol E Baron R Dworkin R . Pharmacotherapy for neuropathic pain in adults: a systematic review and meta-analysis. Lancet Neurol. (2015) 14:162–73. 10.1016/S1474-4422(14)70251-0 25575710 PMC4493167

[6] Gilron I Baron R Jensen T. Neuropathic pain: principles of diagnosis and treatment. Mayo Clin Proc. (2015) 90:532–45. 10.1016/j.mayocp.2015.01.018 25841257

[7] McGee SL Hargreaves M. Exercise adaptations: molecular mechanisms and potential targets for therapeutic benefit. Nat Rev Endocrinol. (2020) 16:495–505. 10.1038/s41574-020-0377-1 32632275

[8] Owen PJ Miller CT Mundell NL Verswijveren S Tagliaferri SD Brisby H . Which specific modes of exercise training are most effective for treating low back pain? Network meta-analysis. Br J Sports Med. (2020) 54:1279–87. 10.1136/bjsports-2019-100886 31666220 PMC7588406

[9] Wu B Zhou L Chen C Wang J Hu L Wang X. Effects of exercise-induced hypoalgesia and its neural mechanisms. Med Sci Sports Exerc. (2021). 10.1249/MSS.0000000000002781 [Epub ahead of print]. 34468414

[10] Zheng K Chen C Yang S Wang X. Aerobic exercise attenuates pain sensitivity: an event-related potential study. Front Neurosci. (2021) 15:735470. 10.3389/fnins.2021.735470 34630022 PMC8494006

[11] Dobson JL McMillan J Li L. Benefits of exercise intervention in reducing neuropathic pain. Front Cell Neurosci. (2014) 8:102. 10.3389/fncel.2014.00102 24772065 PMC3983517

[12] Chou R Huffman LH. Nonpharmacologic therapies for acute and chronic low back pain: a review of the evidence for an American Pain Society/American College of Physicians clinical practice guideline. Ann Intern Med. (2007) 147:492–504. 10.7326/0003-4819-147-7-200710020-00007 17909210

[13] Yadav V Bever CJr Bowen J Bowling A Weinstock-Guttman B Cameron M . Summary of evidence-based guideline: complementary and alternative medicine in multiple sclerosis: report of the guideline development subcommittee of the American Academy of Neurology. Neurology. (2014) 82:1083–92. 10.1212/WNL.0000000000000250 24663230 PMC3962995

[14] Lewis RA Williams NH Sutton AJ Burton K Din NU Matar HE . Comparative clinical effectiveness of management strategies for sciatica: systematic review and network meta-analyses. Spine J. (2015) 15:1461–77. 10.1016/j.spinee.2013.08.049 24412033

[15] Harvey LA Glinsky JV Bowden JL. The effectiveness of 22 commonly administered physiotherapy interventions for people with spinal cord injury: a systematic review. Spinal Cord. (2016) 54:914–23. 10.1038/sc.2016.95 27349607

[16] Rousing R Jensen RK Fruensgaard S Strøm J Brøgger HA Degn JDM . Danish national clinical guidelines for surgical and nonsurgical treatment of patients with lumbar spinal stenosis. Eur Spine J. (2019) 28:1386–96. 10.1007/s00586-019-05987-2 31098717

[17] Hao J Zhu X Bensoussan A. Effects of nonpharmacological interventions in chemotherapy-induced peripheral neuropathy: an overview of systematic reviews and meta-analyses. Integr Cancer Ther. (2020) 19:1534735420945027. 10.1177/1534735420945027 32875921 PMC7476348

[18] Martin R Chimenti R Cuddeford T Houck J Matheson J McDonough C . Achilles pain, stiffness, and muscle power deficits: midportion achilles tendinopathy revision 2018. J Orthop Sports Phys Ther. (2018) 48:A1–38. 10.2519/jospt.2018.0302 29712543

[19] Delitto A George S Van Dillen L Whitman J Sowa G Shekelle P . Low back pain. J Orthop Sports Phys Ther. (2012) 42:A1–57. 10.2519/jospt.2012.42.4.A1 22466247 PMC4893951

[20] Blanpied P Gross A Elliott J Devaney L Clewley D Walton D . Neck pain: revision 2017. J Orthop Sports Phys Ther. (2017) 47:A1–A83. 10.2519/jospt.2017.0302 28666405

[21] Collins N Barton C van Middelkoop M Callaghan M Rathleff M Vicenzino B . 2018 Consensus statement on exercise therapy and physical interventions (orthoses, taping and manual therapy) to treat patellofemoral pain: recommendations from the 5th International Patellofemoral Pain Research Retreat, Gold Coast, Australia, 2017. Br J Sports Med. (2018) 52:1170–8. 10.1136/bjsports-2018-099397 29925502

[22] Shea B Grimshaw J Wells G Boers M Andersson N Hamel C . Development of AMSTAR: a measurement tool to assess the methodological quality of systematic reviews. BMC Med Res Methodol. (2007) 7:10. 10.1186/1471-2288-7-10 17302989 PMC1810543

[23] Crossley K van Middelkoop M Callaghan M Collins N Rathleff M Barton C. 2016 Patellofemoral pain consensus statement from the 4th International Patellofemoral Pain Research Retreat, Manchester. Part 2: recommended physical interventions (exercise, taping, bracing, foot orthoses and combined interventions). Br J Sports Med. (2016) 50:844–52. 10.1136/bjsports-2016-096268 27247098 PMC4975825

[24] Bossard D Remus A Doherty C Gribble P Delahunt E. Developing consensus on clinical assessment of acute lateral ankle sprain injuries: protocol for an international and multidisciplinary modified Delphi process. Br J Sports Med. (2018) 52:1539. 10.1136/bjsports-2017-099007 29351953

[25] Sanz-Paris A Álvarez Hernández J Ballesteros-Pomar M Botella-Romero F León-Sanz M Martín-Palmero Á . Evidence-based recommendations and expert consensus on enteral nutrition in the adult patient with diabetes mellitus or hyperglycemia. Nutrition. (2017) 41:58–67. 10.1016/j.nut.2017.02.014 28760429

[26] Andresen S Biering-Sørensen F Hagen E Nielsen J Bach F Finnerup N. Pain, spasticity and quality of life in individuals with traumatic spinal cord injury in Denmark. Spinal Cord. (2016) 54:973–9. 10.1038/sc.2016.46 27067654

[27] Boldt I Eriks-Hoogland I Brinkhof MW de Bie R Joggi D von Elm E. Non-pharmacological interventions for chronic pain in people with spinal cord injury. Cochr Database Syst Rev. (2014) 11:Cd009177. 10.1002/14651858.CD009177.pub2 25432061 PMC11329868

[28] Gómara-Toldrà N Sliwinski M Dijkers MP. Physical therapy after spinal cord injury: a systematic review of treatments focused on participation. J Spinal Cord Med. (2014) 37:371–9. 10.1179/2045772314Y.0000000194 24621042 PMC4116720

[29] Labruyère R van Hedel HJ. Strength training versus robot-assisted gait training after incomplete spinal cord injury: a randomized pilot study in patients depending on walking assistance. J Neuroeng Rehabil. (2014) 11:4. 10.1186/1743-0003-11-4 24401143 PMC3905290

[30] Harrison R Field T. Post stroke pain: identification, assessment, and therapy. Cerebrovasc Dis. (2015) 39:190–201. 10.1159/000375397 25766121

[31] Costantino C Galuppo L Romiti D. Short-term effect of local muscle vibration treatment versus sham therapy on upper limb in chronic post-stroke patients: a randomized controlled trial. Eur J Phys Rehabil Med. (2017) 53:32–40. 10.23736/S1973-9087.16.04211-8 27598342

[32] Jeon HJ An S Yoo J Park NH Lee KH. The effect of Monkey Chair and Band exercise system on shoulder range of motion and pain in post-stroke patients with hemiplegia. J Phys Ther Sci. (2016) 28:2232–7. 10.1589/jpts.28.2232 27630403 PMC5011567

[33] Wei YH Du DC Jiang K. Therapeutic efficacy of acupuncture combined with neuromuscular joint facilitation in treatment of hemiplegic shoulder pain. World J Clin Cases. (2019) 7:3964–70. 10.12998/wjcc.v7.i23.3964 31832398 PMC6906577

[34] Horsley S Lannin NA Hayward KS Herbert RD. Additional early active repetitive motor training did not prevent contracture in adults receiving task-specific upper limb training after stroke: a randomised trial. J Physiother. (2019) 65:88–94. 10.1016/j.jphys.2019.02.005 30910563

[35] Solaro C Trabucco E Messmer Uccelli M. Pain and multiple sclerosis: pathophysiology and treatment. Curr Neurol Neurosci Rep. (2013) 13:320. 10.1007/s11910-012-0320-5 23250765

[36] Demaneuf T Aitken Z Karahalios A Leong TI De Livera AM Jelinek GA . Effectiveness of exercise interventions for pain reduction in people with multiple sclerosis: a systematic review and meta-analysis of randomized controlled trials. Arch Phys Med Rehabil. (2019) 100:128–39. 10.1016/j.apmr.2018.08.178 30240593

[37] Edwards T Motl R Sebastião E Pilutti L. Pilot randomized controlled trial of functional electrical stimulation cycling exercise in people with multiple sclerosis with mobility disability. Mult Scler Relat Disord. (2018) 26:103–11. 10.1016/j.msard.2018.08.020 30243234

[38] Pilutti LA Edwards T Motl RW Sebastião E. Functional electrical stimulation cycling exercise in people with multiple sclerosis: secondary effects on cognition, symptoms, and quality of life. Int J MS Care. (2019) 21:258–64. 10.7224/1537-2073.2018-048 31889930 PMC6928578

[39] Grubić Kezele T Babić M Štimac D. Exploring the feasibility of a mild and short 4-week combined upper limb and breathing exercise program as a possible home base program to decrease fatigue and improve quality of life in ambulatory and non-ambulatory multiple sclerosis individuals. Neurol Sci. (2019) 40:733–43. 10.1007/s10072-019-3707-0 30659416

[40] Hasanpour-Dehkordi A Jivad N Solati K. Effects of yoga on physiological indices, anxiety and social functioning in multiple sclerosis patients: a randomized trial. J Clin Diagn Res. (2016) 10:Vc01–5. 10.7860/JCDR/2016/18204.7916 27504387 PMC4963747

[41] Young HJ Mehta TS Herman C Wang F Rimmer JH. The effects of M2M and adapted yoga on physical and psychosocial outcomes in people with multiple sclerosis. Arch Phys Med Rehabil. (2019) 100:391–400. 10.1016/j.apmr.2018.06.032 30092206 PMC9105798

[42] Antonini A Tinazzi M Abbruzzese G Berardelli A Chaudhuri K Defazio G . Pain in Parkinson's disease: facts and uncertainties. Eur J Neurol. (2018) 25:917–e969. 10.1111/ene.13624 29520899

[43] Pérez de la Cruz S. Effectiveness of aquatic therapy for the control of pain and increased functionality in people with Parkinson's disease: a randomized clinical trial. Eur J Phys Rehabil Med. (2017) 53:825–32. 10.23736/S1973-9087.17.04647-0 28627861

[44] Diab AA Moustafa IM. The efficacy of forward head correction on nerve root function and pain in cervical spondylotic radiculopathy: a randomized trial. Clin Rehabil. (2012) 26:351–61. 10.1177/0269215511419536 21937526

[45] Halvorsen M Falla D Gizzi L Harms-Ringdahl K Peolsson A Dedering Å. Short- and long-term effects of exercise on neck muscle function in cervical radiculopathy: a randomized clinical trial. J Rehabil Med. (2016) 48:696–704. 10.2340/16501977-2120 27494094

[46] Fritz JM Thackeray A Brennan GP Childs JD. Exercise only, exercise with mechanical traction, or exercise with over-door traction for patients with cervical radiculopathy, with or without consideration of status on a previously described subgrouping rule: a randomized clinical trial. J Orthop Sports Phys Ther. (2014) 44:45–57. 10.2519/jospt.2014.5065 24405257

[47] Fernandez M Hartvigsen J Ferreira ML Refshauge KM Machado AF Lemes Í . Advice to stay active or structured exercise in the management of sciatica: a systematic review and meta-analysis. Spine. (2015) 40:1457–66. 10.1097/BRS.0000000000001036 26165218

[48] Albert HB Manniche C. The efficacy of systematic active conservative treatment for patients with severe sciatica: a single-blind, randomized, clinical, controlled trial. Spine. (2012) 37:531–42. 10.1097/BRS.0b013e31821ace7f 21494193

[49] Deli G Bosnyak E Pusch G Komoly S Feher G. Diabetic neuropathies: diagnosis and management. Neuroendocrinology. (2013) 98:267–80. 10.1159/000358728 24458095

[50] Cox ER Gajanand T Burton NW Coombes JS Coombes BK. Effect of different exercise training intensities on musculoskeletal and neuropathic pain in inactive individuals with type 2 diabetes - preliminary randomised controlled trial. Diabetes Res Clin Pract. (2020) 164:108168. 10.1016/j.diabres.2020.108168 32360399

[51] Win M Fukai K Nyunt HH Linn KZ. Hand and foot exercises for diabetic peripheral neuropathy: a randomized controlled trial. Nurs Health Sci. (2020) 22:416–26. 10.1111/nhs.12676 31876991

[52] Andersen Hammond E Pitz M Shay B. Neuropathic pain in taxane-induced peripheral neuropathy: evidence for exercise in treatment. Neurorehabil Neural Repair. (2019) 33:792–9. 10.1177/1545968319860486 31342880

[53] Tough D Robinson J Gowling S Raby P Dixon J Harrison SL. The feasibility, acceptability and outcomes of exergaming among individuals with cancer: a systematic review. BMC Cancer. (2018) 18:1151. 10.1186/s12885-018-5068-0 30463615 PMC6249900

[54] Hwang JH Chang HJ Shim YH Park WH Park W Huh SJ . Effects of supervised exercise therapy in patients receiving radiotherapy for breast cancer. Yonsei Med J. (2008) 49:443–50. 10.3349/ymj.2008.49.3.443 18581595 PMC2615347

[55] Dhawan S Andrews R Kumar L Wadhwa S Shukla G. A randomized controlled trial to assess the effectiveness of muscle strengthening and balancing exercises on chemotherapy-induced peripheral neuropathic pain and quality of life among cancer patients. Cancer Nurs. (2020) 43:269–80. 10.1097/NCC.0000000000000693 30888982

[56] Parker R Stein D Jelsma J. Pain in people living with HIV/AIDS: a systematic review. J Int AIDS Soc. (2014) 17:18719. 10.7448/IAS.17.1.18719 24560338 PMC3929991

[57] Maharaj SS Yakasai AM. Does a rehabilitation program of aerobic and progressive resisted exercises influence HIV-induced distal neuropathic pain? Am J Phys Med Rehabil. (2018) 97:364–9. 10.1097/PHM.0000000000000866 29189306

[58] Tumusiime DK Stewart A Venter FWD Musenge E. The effects of a physiotherapist-led exercise intervention on peripheral neuropathy among people living with HIV on antiretroviral therapy in Kigali, Rwanda. S Afr J Physiother. (2019) 75:1328. 10.4102/sajp.v75i1.1328 31535052 PMC6739563

[59] Wylde V Dennis J Beswick A Bruce J Eccleston C Howells N . Systematic review of management of chronic pain after surgery. Br J Surg. (2017) 104:1293–306. 10.1002/bjs.10601 28681962 PMC5599964

[60] McNeely ML Campbell K Ospina M Rowe BH Dabbs K Klassen TP . Exercise interventions for upper-limb dysfunction due to breast cancer treatment. Cochr Database Syst Rev. (2010) 6:CD005211. 10.1002/14651858.CD005211.pub2 20556760 PMC12861582

[61] De Groef A Van Kampen M Dieltjens E Christiaens MR Neven P Geraerts I . Effectiveness of postoperative physical therapy for upper-limb impairments after breast cancer treatment: a systematic review. Arch Phys Med Rehabil. (2015) 96:1140–53. 10.1016/j.apmr.2015.01.006 25595999

[62] Ammitzbøll G Andersen KG Bidstrup PE Johansen C Lanng C Kroman N . Effect of progressive resistance training on persistent pain after axillary dissection in breast cancer: a randomized controlled trial. Breast Cancer Res Treat. (2020) 179:173–83. 10.1007/s10549-019-05461-z 31605312

[63] Guyatt G Oxman A Vist G Kunz R Falck-Ytter Y Alonso-Coello P . GRADE: an emerging consensus on rating quality of evidence and strength of recommendations. BMJ. (2008) 336:924–6. 10.1136/bmj.39489.470347.AD 18436948 PMC2335261

[64] Guyatt G Oxman A Akl E Kunz R Vist G Brozek J . GRADE guidelines: 1. Introduction-GRADE evidence profiles and summary of findings tables. J Clin Epidemiol. (2011) 64:383–94. 10.1016/j.jclinepi.2010.04.026 21195583

