# Supplementary material for: Exercise for Neuropathic Pain: A Systematic Review and Expert Consensus
Source: Front Med (Lausanne). 2021 Nov 24;8:756940. doi: 10.3389/fmed.2021.756940 (PMC8654102; doi:10.3389/fmed.2021.756940)
Supplement: Supplementary Material 1 — The details of the search strategy for the PubMed database. [file Table_1.DOCX]

Exercise for Neuropathic Pain: a systematic review and expert consensus

Search Strategy for Pubmed:

#1. Neuropathic Pain[Title/Abstract] OR Neuralgias[Title/Abstract] OR Neurodynia[Title/Abstract] OR Neurodynias[Title/Abstract] OR Nerve Pain[Title/Abstract] OR Neuropathic Pain[mesh] OR Neuralgias[mesh] OR (stroke[Title/Abstract] and pain[Title/Abstract]) OR (spinal cord injury[Title/Abstract] and pain[Title/Abstract]) OR (multiple sclerosis[Title/Abstract] and pain[Title/Abstract]) OR Postherpetic Neuralgia[Title/Abstract] OR Trigeminal Neuralgia[Title/Abstract] OR (Radiculopathy[Title/Abstract] and pain[Title/Abstract]) OR (Polyneuropathy[Title/Abstract] and pain[Title/Abstract]) OR (diabetic neuropathy[Title/Abstract] and pain[Title/Abstract]) OR (peripheral nerve injury[Title/Abstract] and pain[Title/Abstract]) OR (Post amputation[Title/Abstract] and pain[Title/Abstract]) OR sciatica[Title/Abstract] OR (HIV[Title/Abstract] and pain[Title/Abstract]) OR (Parkinson[Title/Abstract] and pain[Title/Abstract])

(44,624)

#2. exercise[Mesh] OR "Muscle Stretching Exercises"[Mesh] OR "Endurance Training"[Mesh] OR Resistance Training[Mesh] OR exercise[Title/Abstract] OR training[Title/Abstract]

(751,879)

**#3 #1 and #2 (746)**

Search time: 25/01/2021
